# Performance Evaluation Analysis of Spark Streaming Backpressure for Data-Intensive Pipelines

**DOI:** 10.3390/s22134756

**Published:** 2022-06-23

**Authors:** Kassiano J. Matteussi, Julio C. S. dos Anjos, Valderi R. Q. Leithardt, Claudio F. R. Geyer

**Affiliations:** 1 Institute of Informatics, Federal University of Rio Grande do Sul, UFRGS/PPGC, Porto Alegre 91501-970, RS, Brazil; kjmatteussi@inf.ufrgs.br (K.J.M.); geyer@inf.ufrgs.br (C.F.R.G.); 2 LIG-ERODS, Université Grenoble Alpes, 38058 Grenoble, France; 3 Graduate Program in Teleinformatics Engineering Federal, University of Ceará, PPGETI/UFC, Center of Technology, Campus of Pici, Fortaleza 60455-970, CE, Brazil; jcsanjos@ufc.br; 4 COPELABS, Universidade Lusófona de Humanidades e Tecnologias, 1749-024 Lisboa, Portugal; 5 VALORIZA, Research Center for Endogenous Resource Valorization, Polytechnic Institute of Portalegre, 7300-555 Portalegre, Portugal

**Keywords:** backpressure, big data, spark streaming, stream processing

## Abstract

A significant rise in the adoption of streaming applications has changed the decision-making processes in the last decade. This movement has led to the emergence of several Big Data technologies for in-memory processing, such as the systems Apache Storm, Spark, Heron, Samza, Flink, and others. Spark Streaming, a widespread open-source implementation, processes data-intensive applications that often require large amounts of memory. However, Spark Unified Memory Manager cannot properly manage sudden or intensive data surges and their related in-memory caching needs, resulting in performance and throughput degradation, high latency, a large number of garbage collection operations, out-of-memory issues, and data loss. This work presents a comprehensive performance evaluation of Spark Streaming backpressure to investigate the hypothesis that it could support data-intensive pipelines under specific pressure requirements. The results reveal that backpressure is suitable only for small and medium pipelines for stateless and stateful applications. Furthermore, it points out the Spark Streaming limitations that lead to in-memory-based issues for data-intensive pipelines and stateful applications. In addition, the work indicates potential solutions.

## 1. Introduction

Stream Processing (SP) is a trending topic that represents a remarkable milestone for data-intensive processing and analysis in both industry and research fields [1,2]. Moreover, SP systems have provided near or real-time data analysis for numerous network-based applications and services in the most varied areas and domains, such as financial services, healthcare, education, manufacturing, retail, social media, and sensor networks [3,4].

Nonetheless, the considerable growth of distributed frameworks for the most varied purposes of Big Data analytics, such as Apache Storm [5], Samza [6], Apache Spark [7], Flink [8], Amazon Kinesis Streams [9], and others, is noticeable. These frameworks were designed to enable flexible solutions to persist and process data-intensive workloads in memory [10]. In addition, the memory processing minimizes disk I/O movements, reduces the data processing time significantly, and outperforms the well-established Hadoop MapReduce implementation [7].

Spark Streaming (SS), for instance, provides iterative in-memory data processing with low latency by using the Resilient Distributed Datasets (RDD) abstraction. RDD represents the distributed data blocks organized into small partitions to maximize parallel processing. The RDD processing and cache rely on Spark Unified Memory Management (UMM), which dynamically manages data execution and storage regions in the executor Java Virtual Machine (JVM) heap. The execution region from Spark supports runtime processing operations such as shuffle, join, sort, and aggregation. On the other hand, the storage region caches RDD data blocks for both current processing and re-processing tasks, as well as storing the incoming data to be further processed [11].

However, Spark could present performance degradation due to a lack of memory management for very intensive and dynamic memory-borrowing operations between execution and storage regions at the UMM level [10,11]. It occurs because processing data overflow is costly for the UMM execution region space, requiring heap space from the storage region under pressure conditions. However, the UMM storage region will keep caching incoming data during the whole processing life cycle, resulting in very dynamic borrowing operations between the regions.

This scenario could be even worse since UMM gives a higher priority to the execution memory than to storage memory [12]. Therefore, execution and storage area overloading will lead to several implications, such as significant recomputing overhead, unnecessary data block eviction, long and numerous Garbage Collection (GC), Out Of Memory (OOM) exceptions, throughput degradation, high processing latency, data loss, and memory contention.

Previous studies reveal that resource management for Big Data analysis concerns both batch and stream processing systems [13,14,15,16,17,18,19]. Therefore, extending this problem to other SP systems, such as Flink and Storm, can help the JVM data processing and storage operation support by using varied data-persisting approaches such as on-heap, disk only, and off-heap. The common point of these approaches with Spark is the limited support for data-intensive caching operations due to the restricted size of JVM heap space, disk, or other combinations. In addition, the complexity behind the configuration of each approach is hard to manage.

Furthermore, it is noticeable that data pipelines for SP could produce data faster than the downstream operators can consume, requiring large amounts of memory [20]. In such a case, backpressure [21] mechanisms have been widely adopted in the most varied domains of SP systems. This mechanism helps applications to keep data processing under control by managing data ingestion and processing rates. Nevertheless, the backpressure reacts to the processing needs for a graceful response to sudden and intensive loads of data rather than facing a system crash [21].

This work proposes a performance evaluation of Spark Streaming backpressure to investigate the hypothesis that it could support data-intensive pipelines under pressure conditions. The investigation is guided by real-world evaluation scenarios comprising stateful and stateless applications on top of modern-hardware architectures. The contributions are three-fold:

(i) It provides a deep dive into SS backpressure, its underlying components, and in-memory management needs for data-intensive pipelines;
(ii) This paper proposes a performance evaluation with a data-streaming-intensive approach similar to real production scenarios. Still, the assessment and remarks of this study point out varied performance insights that may contribute to SP communities to create more accurate and robust in-memory solutions for SP systems;
(iii) This work reveals a current limitation for both SS and its backpressure system for supporting data caching operations in data-intensive SP pipelines. In addition, it demonstrates that the constraints may affect SS and other SP systems in providing in-memory data processing and analysis.

The remainder of this paper is organized as follows. Section 2 presents the literature review and shows related work discussions. Section 3 presents SS backpressure, its model, architecture, and in-memory management needs for SP systems. The materials and methods description is shown in Section 4. Section 5 presents the SS evaluation, results, and insights. Finally, Section 6 presents the conclusions.

## 2. Literature Review

An essential requirement of SP systems is robustness against variations in streaming workloads. For example, the SP should adapt quickly to sudden spikes in workload demands. This section investigates how SP systems handle incoming data from varied streaming sources without degrading applications’ throughput. Still, this section aims to understand the existing solutions and their current limitations to point out opportunities regarding memory management for data-intensive SP pipelines.

Das, T. et al. [22] presents an adaptive batch sizing strategy for SP systems. It is based on a fixed-point iteration solution, a well-known numerical optimization technique that allows the system to adapt the window size when incoming data vary too much dynamically. Thus, it is possible to minimize end-to-end latency while keeping the system stable based on the statistics of the last two completed batches. This strategy allows for the better use of resources since it avoids high processing delays and load spikes, which lead the SP system to build up batches in memory, and results in a low throughput performance and system crashes.

However, the solution does not present related-memory management strategies that directly impact the memory governance task. It prototypes an end-to-end controller that introduces data orchestration and load balancing using a batching strategy. Still, it can be considered a promising solution that introduces the queue concept widely adopted by Message Queue (MQ) systems. Thus, a MQ coordinator could help to orchestrate the incoming data between up and downstream operators, avoiding OOM issues in data-intensive SP pipelines.

Birke, R. et al. [23] proposed a data-driven latency controller that estimates how many data can be processed in a single time window. The solution is based on performance metrics obtained from Spark execution, such as Scheduling Delay (SD) and Processing Time (PT). Then, for each time step, the solution will measure the current SD and PT to define the new processing rate. Still, if the incoming data overflow the SP system capacity, a shedding threshold will be set to drop data out. Then, new data blocks will only be accepted if they fit into the current time window. Otherwise, they will be dropped out by the shedding strategy to avoid high load spikes.

This work is quite similar to the current backpressure mechanism allowed by Spark. However, the major drawback of this work relies on the data-shedding strategy. Thus, even decreasing processing delays, the solution does not avoid data loss and may obtain the worst results in data-intensive SP pipelines. Still, this solution ignores the memory utilization from Spark or related-memory strategies that directly impact the processing performance.

Chen, Xin. et al. [24] presented a checkpointing feedback controller as a complementary mechanism to act alongside Spark backpressure to manage the checkpointing time. It was made to achieve a solid execution and a high throughput, similar to Proportional-Integral-Derivative (PID) schema in the Spark framework. The solution collects historical data such as SD and PT from past batch jobs between a set of checkpoints. In this case, the author defines one region as a collection of ten seconds (ten jobs), where nine are set for processing, and one for the checkpoint. Then, based on the retrieved information from processing, it is possible to measure the number of tuples for processing and minimize the data ingestion of the next jobs to decrease the delay cost associated with the checkpoints task gradually. It represents a similar behavior to the one applied by PID to Spark receivers.

Spark allows for the use of the *.timeout()* (State timeout: https://spark.apache.org/docs/2.0.0/api/java/org/apache/spark/streaming/State.html, accessed on 19 May 2022) function to control state checkpointing persistence. In such a case, it is highly recommended to specify this feature for data-intensive SP applications. Otherwise, the state checkpoint becomes bigger, and the system could run out of memory. Although the author implemented a module to collect information, Spark also allows for the use of a well-defined listener interface such as *onBatchCompleted()* (Spark listeners: https://spark.apache.org/docs/2.1.0/api/java/org/apache/spark/streaming/scheduler/StreamingListener.html, accessed on 19 May 2022) for receiving information about an ongoing streaming computation. Finally, although it lets the processing become under control, this solution ignores the incoming data and related use from memory at the Spark level, directly impacting the processing performance.

Hanif, Muhammad et al. [21] present a backpressure mitigation mechanism for in-memory data SP frameworks. This study reveals how Flink’s backpressure is propagated in the opposite direction of downstream operators. It means that backpressure is not fully aware of the operator performance, and it may affect the performance due to memory management problems at the JVM level. Still, the upstreams may produce data faster than the downstream operators can consume, overloading JVM memory.

In such a context, the proposed strategy focuses on stateful applications and aims to improve its performance by adjusting the level of parallelism of each operator on the fly. Thus, a feedback loop was made to identify whether the data ingestion is faster than the downstream operators can consume. This strategy uses a ratio-based algorithm that measures Central Processing Unit (CPU) utilization to set up a ratio value to establish the current sensibility of processing. The ratio varies from 0 to 1 and indicates the current system’s condition. For instance, a value near zero does not represent a backpressure scenario, but a valuer near 1 indicates a backpressure one. Based on the ratio value, it is possible to increase or decrease the level of parallelism from operators on the fly in order to alleviate the buffer overflow.

Although Flink has been taking advantage of several Application Programming Interface (API)s, such as backpressure, to help in task performance management, this work does not measure memory utilization from the operators, and it may lead to incorrect decisions. Still, as the authors described, the OOM should occur in extreme conditions without controlling incoming data, thus leading to a memory starvation problem. Unfortunately, the author provided a narrow evaluation that does not comprise a real-world case scenario and leads the system to a bad state. Finally, this work reinforces the need for a global controller to keep incoming data under control. At the very least, Flink and Spark rely upon JVM for execution overflow and manage OOM issues by spilling data to the disk, degrading the performance.

De Souza. Paulo et al. [15] introduce BurstFlow, a tool for enhancing communication across data sources located at the edges of the Internet and Big Data SP applications located in cloud infrastructures. BurstFlow introduces a strategy for adjusting the micro-batch sizes dynamically according to the time required for communication and computation. In addition, it presents an adaptive data partition policy for distributing incoming data across available machines by considering memory and CPU capacities. This approach leads to overcoming resource contention scenarios while maintaining network stability. Real-world experiments show an improvement of over 9% in the execution time, which is over 49% better CPU and memory utilization compared to methods applied to data partitioning in Apache Flink and the state of the art.

The up-streams components can produce data faster than the downstream operators can consume, thus overloading JVM memory. The author proposes a dynamic strategy to batch data that maximizes the throughput and minimizes network latency over heterogeneous environments. However, there is a scheduling and data imbalance problem, leaving JVM free to keep receiving data, even in intensive conditions.

The authors in [20] mention that a Big Data system such as Spark is highly memory-intensive. This work reinforces that a lack of memory can lead to several functional and performance issues, including OOM crashes, a significantly degraded efficiency, or even a loss of data upon node failures. The author investigates the performance of dynamic random access memory and non-volatile memory and argues that this kind of memory is not yet fully explored. In such a context, GC represents a challenge since it must lead with applications written in Java and Scala that are executed on top of the JVM.

However, JVM is not aware of hybrid memories and computing needs. Thus, during RDD processing, GC copies objects for varied physical memory pages, which breaks the bonding between data and physical memory address, leading to interference in memory management. The authors propose Panthera to manage data processing in accordance with the semantics of applications and infers the coarse-grained data usage behavior by light-weight static program analysis and dynamic data usage monitoring. Panthera leverages garbage collection to migrate data between dynamic random access memory and non-volatile memory, incurring a low overhead.

It is well known that the available memory of computing systems is constantly increasing in order to allow for in-memory data processing at a high scale. Moreover, in-memory data-intensive SP frameworks have been widely used to handle challenging problems in various domains, such as machine learning, graph computing, and SP. Thus, the applications have been benefiting from in-memory operations, since using them is faster than accessing the disk or receiving data from the network. Table 1 summarizes the literature review and points out the main problems, memory characteristics, related issues, and strategies applied in data-intensive SP scenarios.

As far as we can see, the goal of finding an efficient memory management solution has become key for allowing high-performance processing in the most varied areas and domains. It has become a concern for streaming stateful and stateless applications, since the solutions’ design is not agnostic of the environment or applications, and depends on core modification in SP systems. Unfortunately, SP systems hide the memory management scheme (data persistence, e.g., memory, disk, and other combinations) from users who do not have the opportunity to monitor and configure the memory resources properly. For instance, the control of the JVM or internal mechanism from frameworks such as Spark UMM; the current cache replacement policies based on the Direct Acyclic Graph (DAG), such as Spark Least Recently Used (LRU), which do not consider the dynamic change in cache capacity needed by data-intensive SP applications; and so on. Both cases may lead to data blocks being evicted, producing significant recomputing overhead or data loss.

## 3. Spark Backpressure: Model and Architecture

Spark backpressure provides a dynamic management of executor processing rates according to the PT and SD application performance metrics. It uses the PID controller as core implementation, also known as the three-term controller created by Ziegler–Nichols and Nathaniel B. [25]. PID implements a feedback loop mechanism widely used in the industry to decrease the complexity behind the configuration of dynamic control systems such as the behavior found in data-intensive SP systems. The backpressure model implementation includes the Spark performance counters, as seen in Table 2 in the PID model.

**Figure 1:**
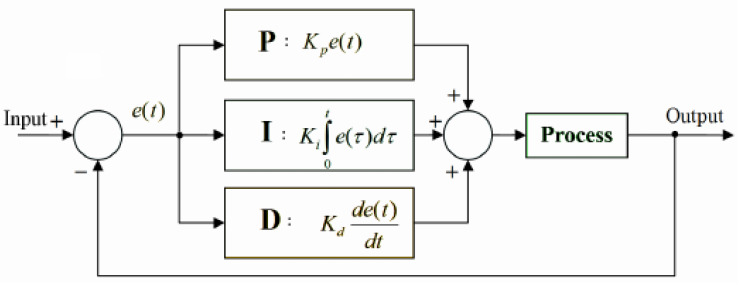
PID Controller Model Implementation.

Figure 1 presents the PID controller implemented by Spark. The goal of the PID model is to automatically calculate processing rates to be applied in the Spark executors continuously.

The main components of PID controller implementation (PID implementation: https://github.com/apache/spark/blob/master/streaming/src/main/scala/org/apache/spark/streaming/scheduler/rate/PIDRateEstimator.scala, accessed on 19 May 2022) are:

- Error e(t): It represents the number of records that overloaded the defined time window from a given batch (Process). It is used as the basis for PID measurements;
- Proportional (P): Term *P* is proportional to the current obtained error e(t), and this value can be positive or zero, being proportionately adjusted, taking into account that a gain factor value *d*-default value is 1. If there is no error, there is no corrective response. At the Spark-level, $K_{p}$ is defined by *spark.streaming.backpressure.pid.proportional* (default weight: 1 non-negative) and represents the current error over the PT rate e(t);
- Integral (I): The *I* value is measured based on the past values and is integrated on-the-fly to produce the output. For instance, if there is a residual error e(t) from proportional control, the integral tries to eliminate this residual error by applying a weight-based factor. If the error is minimized, the *I* term will be proportional to the error decreases. At the Spark-level, $K_{i}$ is defined by *spark.streaming.backpressure.pid.integral* (default weight: 0.2, non-negative) and it is well known as a historical error that uses the current SD as an indicator for the overflow elements that could not be processed in previous batches;
- Derivative (D): The *D* term represents an estimation related to the current rate of change. It is also called “anticipatory control” because it reduces the effect of error e(t) by rating the error change rate. If the state changes quickly, this value reacts at the same rate, controlling or damping the effect. The value is kept equal to zero in the Spark since the controller does not expect abrupt oscillation in the processing rates.

In summary, the new processing rate represents the error value e(t) obtained by the difference between a desired rate calculated for the previous output batch (y(t)) and a measured rate obtained for the current input batch (r(t)), where r(t) represents the number of processed events divided by the time window, resulting in e(t)=r(t)−y(t). As indicated in Figure 1, Spark covers $K_{p}$ for reacting to the error proportionally; for instance, how much the correction should depend on the current error. If the value chosen for this term is considerable, the controller could overshoot the established point, and a small one makes the controller too insensitive. At the same time, $K_{i}$ measures how much the correction should depend on the accumulation of past errors. Thus, $K_{i}$ accelerates a movement towards the desired value. However, an enormous value may lead to overshooting. The $K_{d}$ term provides some speculation to seek how much the correction should depend on predicting future errors based on the current rate of change. Usually, $K_{d}$ is not used very frequently since it can impact the SP system stability. Finally, the PID controller presents an exciting property that lacks a guarantee of a stable fixed point [26]. Due to its nature, the algorithm will oscillate around a (near-optimal) constant throughput until steady.

**Figure 2:**
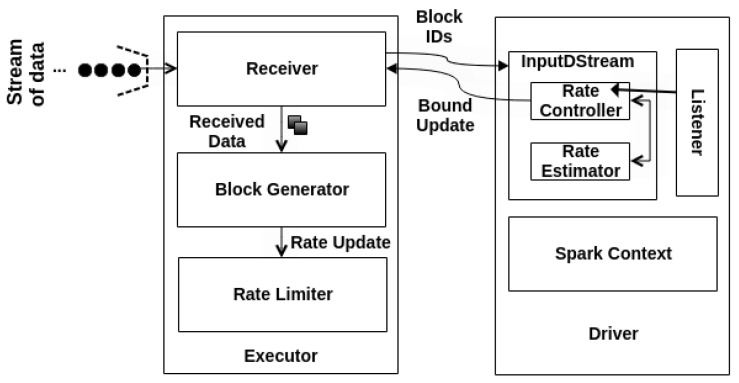
Spark Backpressure PID Architecture.

Behind the hype, Spark implements some architectural components to support the PID model, such as Rate Controller (RC), Rate Estimator (RE), and Rate Limiter (RL). Figure 2 presents the Spark backpressure PID architecture. Indeed, RC and RE components execute side by side to deliver the maximum processing speed on the Driver side. At the same time, the RL updates the maximum processing rate into the executors right after the Driver’s notification update.

The RC (Rate Controller Implementation: https://github.com/apache/spark/blob/master/streaming/src/main/scala/org/apache/spark/streaming/scheduler/RateController.scala, accessed on 19 May 2022) represents a contract for a single Dstream that grabs information from Spark listener for every batch completed to measure the current processing rate and then estimates a new one [27]. The DStream abstraction allows for the processing of the continuous data stream that comprises a RDDs’ sequence.

The listener *onBatchCompleted* receives periodic information from all batch jobs, such as the current number of processed events, PT, and SD. Then, this information will be taken by PID implementation in RE (Rate Estimator Implementation: https://github.com/apache/spark/blob/master/streaming/src/main/scala/org/apache/spark/streaming/scheduler/rate/RateEstimator.scala, accessed on 19 May 2022) to estimate a reasonable data processing rate. The RE could have multiple-way implementations, but this work adopts the PID-based version as standard.

Moreover, the PID controller only computes a new rate if there are events to process. Otherwise, it skips the rate estimation and does not return any rate limit. Then, RE stores the new rate and forwards it to RL. RL sets the maximum rate of events to not exceed the current load, usually by assigning a value lower than the last established rate. The new bound is related to the number of events that the receivers will allow for processing per second at the executor level.

### 3.1. Spark Streaming Memory Management

Spark allows for the use of the UMM to provide dynamic allocation for both execution and storage areas. Thus, if the storage or execution environments receive insufficient spaces, the memory region will be handled according to a dynamic occupancy mechanism [11]. Finally, Spark releases JVM for execution and storage. Thus, it uses GC and LRU [28] mechanisms to clean old objects and blocks from memory.

**Figure 3:**
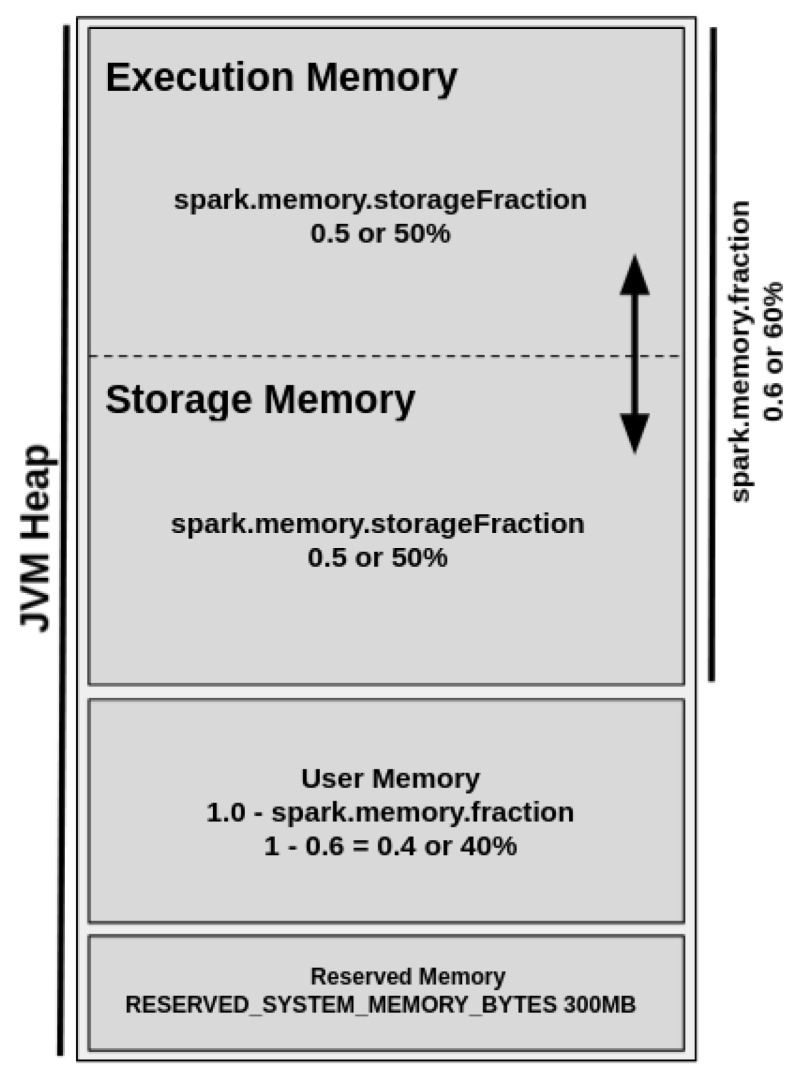
Unified Memory Manager.

The UMM was introduced in Spark 1.6 to replace the SMM model. The UMM allocates execution and storage as a unified memory region that provides dynamic memory management. Thus, when execution memory is not used, the storage memory could acquire all of the available memory, and vice versa [11]. Figure 3 shows the UMM diagram.

By default, the Reserved Memory is hard-coded, and its size should be at least 1.5 times of reserved memory size and cannot be changed in any way without Spark recompilation or by changing the configuration of *spark.testing.reservedMemory* setting. The User Memory represents 40% of JVM heap memory and stores user-defined data structures and functions, Spark internal metadata, data needed for RDD operations, and all of the information regarding RDD dependency and others. It is also commonly used as a “backup” to prevent OOM issues.

The Spark Memory Fraction represents 60% of JVM heap memory and unifies the regions responsible for the management of all cached, persisted, or other intermediate data. This region is allowed by the configuration of the configuration of *spark.memory.storageFraction* parameter and enables the management of storage and execution sub-regions.

The storage region represents 50% of the Spark Memory Fraction region and aims to store all cached data, broadcast variables, unroll process (deserializing data contained in RDD partitions), and so on. On the other hand, the execution region allows for shuffle buffering and stores all of the objects required during the execution of Spark tasks and related operations, such as shuffle, join, sort, and aggregations. Still, the execution region can represent 50% of the Spark Memory Fraction region and could be defined as more “short-lived” than the storage one because the data blocks could be evicted from JVM heap memory immediately after each operation, making space for new computation. However, even in intensive conditions, these data blocks cannot be forcefully evicted by other running threads (tasks).

### 3.2. Spark Cache Management Problems

**Figure 4:**
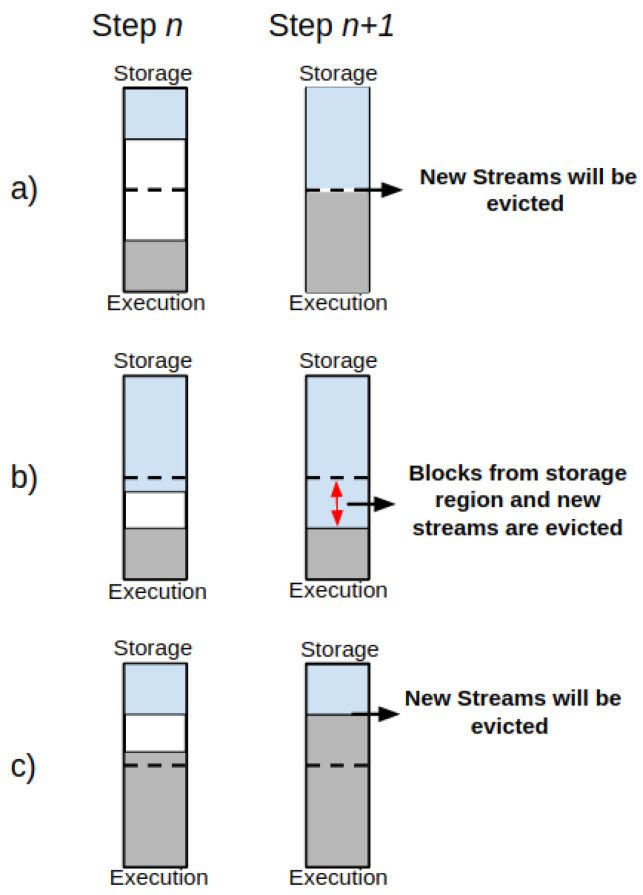
Memory Management Behaviour.

Efficient memory allocation is a critical task for performance in SP systems. In-memory processing frameworks such as SS should allow for data-intensive processing for various applications. However, Spark could present performance issues due to a lack of memory management for very dynamic and intensive operations at the UMM level. In such a case, the RDD data blocks could be evicted from the UMM storage memory region or even neglected before processing. Consequently, the execution can suffer from several OOM exceptions, job and system crashes, data loss, throughput degradation, and high latency. Figure 4 presents the memory management behavior of the UMM’ storage and execution regions.

**Figure 5:**
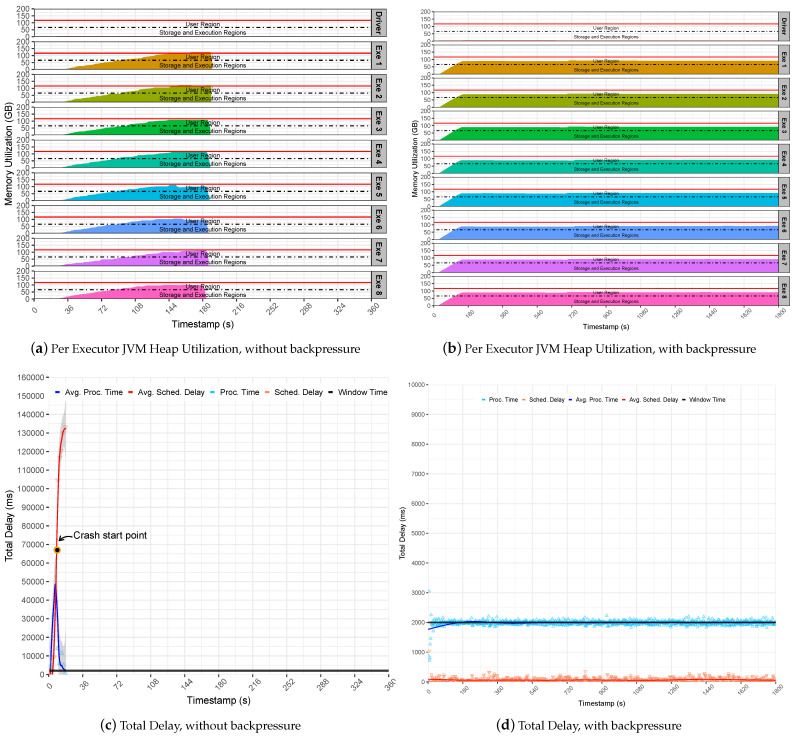
Stateless SumServer Application-Pipeline 2-Parasilo Cluster.

In such a case, it is possible to find the following behaviors:

(1) The storage region can borrow space from the execution one only if blocks are not used in the execution region;
(2) The execution region can also borrow space from the storage one if blocks are not used in storage memory;
(3) If space in both execution and storage regions is insufficient, then the new stream of data will be spilled off—see Figure 4a;
(4) If the storage region borrows space from the execution region and the execution region claims it back, the data blocks from the storage region in the borrowed space from execution will be evicted gradually until the established limit for the storage region gets reached, and then the new streams of data will be spilled off until this procedure ends—see Figure 4b;
(5) If the execution region borrows space from the storage region, but the storage region claims it back, then the new streams of data will be spilled off while the execution region releases the memory space from storage. See Figure 4c.

Usually, JVM enables user memory to prevent the OOM issues, as described earlier in this section. However, the eviction process may also occur between memory-borrowing operations since the memory is under pressure due to excessive use from UMM regions. In practice, the UMM design avoids any modification of runtime operations in the execution memory because they are costly for performance [29].

Still at the UMM level, Spark provides the LRU strategy to help with the task of DAG-dependency eviction by removing old cached objects. In such a context, recent studies proposed LRU-based solutions, with noticeable improvements in comparison to the Spark LRU strategy [10,11]. However, the main observations highlight that a lack of flexibility and related priority is given to the execution over the storage region from UMM.

In summary, the JVM heap may represent a point of failure during application processing due to a high memory consumption at the UMM level, leading Spark to an under-pressure scenario. Consequently, executors could spend a long processing time due to the numerous GC operations, causing long processing delays and system crashes. Therefore, the less memory space that the RDD takes up in the executors’ JVM heap memory, the more heap space that will be available for application processing, thus increasing the GC efficiency. Otherwise, excessive memory consumption by RDD leads to a significant performance loss (shortened by 10% or 20%) due to a large number of buffered objects at the heap level [30].

## 4. Materials and Methods

This section describes the materials and methods applied to experiments investigating how Spark’s Streaming backpressure performs in data-intensive pipelines. The experimental setup used at this work relied on an open-source French Grid5000 (Grid5000 hardware specification is available at: https://www.grid5000.fr/w/Hardware, accessed on 19 May 2022) consortium that provides a modern hardware environment for experimental purposes. The environments chosen for the experiments were Parasilo cluster in Rennes and Dahu cluster in Grenoble. In both cases, the processing scenarios comprised at least a minimum set of 18 nodes and a maximum set of 25 nodes per cluster in total, varying only in the number of MQs set for each experiment. The hardware of the clusters is presented as follows.

- Dahu cluster uses Dell PowerEdge C6420 nodes comprising Intel Xeon Gold 6130 (Skylake, 2.10 GHz, 2 CPUs/node, sixteen cores/CPU)—64 cores in total (hyper-threading), 192 GB of memory, two interfaces (i) 10 Gbps, model: Intel Ethernet Controller X710 for 10GbE SFP+, and (ii) Omni-Path, 100 Gbps, model: Intel Omni-Path HFI Silicon 100 Series;
- Parasilo cluster uses Dell PowerEdge R630 nodes comprising Intel Xeon E5-2630 v3 (Haswell, 2.40 GHz, 2 CPUs/node, eight cores/CPU)—32 cores in total (hyper-threading), 128 GB of memory, two 10 Gbps network cards interconnected with a 10 G Nexus 56128P network switch.

Each setup allows for the use of the software stack presented in Table 3.

In addition, both SS and HDFS used a RAMFS mounted folder to write shuffle data and checkpoints. The RAMFS also supports all installation folders, such as data and name nodes of Hadoop, Spark, or even logs of applications such as GC, or monitoring tools such as dstat monitor such as Dstat-based monitor tool available at ( https://github.com/mvneves/dstat-monitor, accessed on 19 May 2022). In addition, at the Spark side, the monitoring and user interface were deactivated to avoid extra communication costs or any level of interference in the experiments. At the configuration level, the proposed evaluation will consider real-world guidelines, presented as follows.

- Spark could run by using a minimum of 8 GB to hundreds of gigabytes of memory per machine. In general, the recommended memory for Spark Workers represents an allocation of at least 75% to Spark, leaving the rest for the OS [31]. The proposed testbed pushes this limit to use up to 90% of available memory to observe the behavior in OS as well;
- JVM does not always behave well with more than 200 GB of RAM. In this case, it is recommended to launch multiple executors per worker node and balance all available resources, such as memory and cores [31]. At this work, this limit was not reached, and each worker in the Spark cluster will host only one Executor that will receive all of the available resources from the given node. Still, the Executor will host only a single receiver;
- The communication must rely on high-speed and low-latency networks, such as 10 gigabit switches or higher, to allow for data processing at scale [31]. The cluster used in this work provided 10 gigabit switches;
- Although SS allows for the use of varied data persisting approaches, this work will make use of on-heap JVM approach to handle both data processing and storage at memory level only;
- G1GC is the low-pause, server-style generational garbage collector for Java HotSpot VM. It is indicated by Spark to improve performance in bottleneck scenarios [32]. This work keeps this standard as a default configuration.

Table 4 summarizes the configuration set for Spark in both clusters. The batch interval represents the window time (2000 ms) in which Spark will receive and process data. The RDD block generation happens every 400 ms. The values chosen reflect a near-real time processing in which the applications could process data at high-scale. All of the experiments were performed using only one DAG in order to observe how master process behaves in the driver node. This work kept default parallelism of Spark since it follows the number of partitions in RDDs, and, in this case, we optimized the partitioning to fit with the number of the available cores in the cluster only for the application Statefull SumServer. Thus, it is possible to extract the maximum performance, pushing up memory utilization, as happens in real-world use cases. Finally, the JVM heap set follows 90% of the available memory per node, and the table also shows the conceptual values from the Spark UMM regions.

Table 5 presents the pipeline configurations used in the evaluations. Two configurations were defined to push data processing until the limit. The idea was to obtain a better understanding of the current limitations from SS surrounding data SP in scenarios with and without a backpressure mechanism. Pipeline 1 represents a soft-sized pipeline that uses a single MQ for data forwarding. Pipeline 2 represents a high-sized pipeline that increases the number of of MQs up to eight in data forwarding layer, introducing a data-intensive approach.

It is necessary to point out some observations regarding the pipeline configurations and its usage. This work aimed to investigate and understand how backpressure works in intensive conditions. For this reason, some rules were adopted: (i) the cluster will be not shared; (ii) the experiments will be made in low-latency and high-speed network connections; (iii) Spark processing should be performed in a non-intrusive environment. In this case, each worker node hosts only one executor, each executor will comprise a single receiver, and each executor will receive all available resources from such worker node.

For all experiments, a data-intensive scientific simulation called Stencyl was used to generate numeric data at scale by using Message Passing Interface (MPI). The Stencyl represents a synthetic implementation that generates data based on a heat distribution simulation in a 2D domain reliant on the Jacobi iterative method. The progression of the simulation was recorded as synchronous time steps. At the end of a time step, the simulation data were forwarded to the MQ. The messages were configured, comprising 10,000 floating points elements (approximately size of 10 KB per message) per MPI rank for each application time step. The MPI ranks were set to 256 in the Parasilo cluster and 512 (representing the number total of processing cores per cluster) for the Dahu cluster, representing simultaneous queues connected to the MQ to maximize the usage of resources during the execution of an application.

The flow of messages is intensive and continuous to keep data communication and processing at high rates. However, if any sending operation fails, the messages are added to a buffer in the respective queue. Still, if the queue buffer is full, the application starts the re-transmission phase, avoiding any data loss. In addition, ZMQ provides I/O threads that take care of the message transmission, thus immediately unlocking the MPI process for computations.

In the current implementation of ZMQ, the ZMQ buffer was set equal to 1000 (default value), and the number of I/O threads was the same number of CPU cores for each MQ node. It is essential to mention that one I/O thread can support at least one gigabyte of data in or out per second [33]. Still, the MQ layer uses intermediary nodes in which its amount depends on the system’s configuration, as presented at Table 5. Those intermediary nodes received messages from *N* simulation processes and retained them in-memory into an input queue buffer before forwarding them to *M* Spark receivers in the executors.

At the consumer level, a Stateless and Stateful SUMServer application was provided to measure a mean computation of scientific simulation data, ensuring all MPI ranks from a given time step into a window will be considered in the current analysis. This application uses a Dstream concept where the RDD is independent and no data are kept between two time windows.

In such a case, the main difference between the applications is the use of states. Thus, to keep information from one RDD to another, a state operator must be used in Statefull application. The state operator is a memory-based element that synchronizes a state over a distributed file system between two time windows. The state operator associates states’ information to a key-value pair. The behavior of the state operator must be manually written. Hence, the first time a key-value pair is met, the state is created. When meeting the key again, the user can update the state value.

In this context, data must be removed manually from the state operator to avoid memory overflow. Thus, the implementation focused on *mapWithState()* function because it provides better performance, being able to provide 8x lower latency (i.e., processing time) and having the capacity to maintain 10x more keys than *updateStateByKey()* [34]. Furthermore, this function allows native support for state timeouts by the *isTimingOut()* function. Then, it is possible to define the period to hold the states in memory before checkpointing. In this work, each state remained in memory for timeout (new Duration (20,000)) ms.

Furthermore, this paper proposes four testing scenarios. The first test case evaluates how stateless applications behave in data-intensive SP pipelines under pressure conditions with and without SS backpressure. The stateless applications are less intrusive for in-memory management since the processing with states is not applied. Following this, this work proposes two evaluations with stateful-based applications. Stateful applications are highly intensive and dynamic for memory, and it may lead the SP system to fail due to OOM issues. Thus, the goal is to evaluate how this kind of application behaves in data-intensive SP pipelines under pressure conditions with and without SS backpressure too. Finally, this work presents a GC comparison analysis to demonstrate how the heap memory performs in intensive conditions in both scenarios with and without Spark streaming backpressure.

The execution time for each experiment was defined as a minimum of 1800 s. The evaluation will present only a subset of the numerous experiments performed to validate our assumptions. Finally, the measured metrics in the experiments were PT, SD in milliseconds, timestamp in seconds, and throughput in megabytes per second (MBps).

## 5. Results and Analysis

This section evaluates if Spark Streaming (SS) backpressure could support data-intensive pipelines under pressure conditions. The Spark configurations and the pipelines used in these experiments are presented in detail in Table 4 and Table 5, respectively. The results will be described in two main evaluation scenarios. Section 5.1 will cover the stateless analysis and compare a backpressure-based and a non-backpressure analysis for the application SUMServer. Section 5.2 extends the previsions stateless evaluation by providing a Stateful-based analysis that aims to investigate in-memory processing issues using two varied essays with and without backpressure, respectively.

### 5.1. Stateless-Based Application Performance Evaluation

This section summarizes the behavior obtained by executing the Stateless SumServer application with and without SS backpressure over Pipelines 1 and 2. Usually, the Stateless applications do not load states in memory and, due to this reason, it is less intrusive for the memory manager. The preliminary study evaluated this application over Pipeline 1 without backpressure. However, as expected, this pipeline was not sufficiently intensive. As a result, both clusters did not forward data to the memory limits, obtaining a PT average lower than the established time window without showing crashes; due to this, it is not represented here.

In contrast, Pipeline 2 revealed that the execution of the Stateless Server application failed suddenly. This happens because the pipeline is intensive and generates a sudden surge of data to Spark executors. This behavior overflows the executors cache that starts to queue up batches in memory for processing. Thus, the SD increased rapidly up to two minutes and pushed PT up to more than one minute, as demonstrated at the note crash start point in Figure 5c. As a result, the Spark suddenly fails, and the master gets overwhelmed with data and stops communicating with the executors, which raises exception messages that require data blocks already evicted from memory. Finally, executors keep caching incoming data and processing residual data until their JVM heap gets full of data and crashes due to OOM issues, as observed in Figure 5a. Even a single executor may lead to a pipeline collapse in the case that it runs out of memory. To notice this observation, inspect the memory utilization and the red lines for each executor row.

On the other hand, the backpressure could support the intensive rates generated by the Pipeline 2, keeping the Stateless SumServer application steady on the defined window time. Still, the stability provided by the backpressure model is noticeable; see Figure 5d. This is because the application presents low SD levels that are steady in less than two ms on average. This results in a stable utilization of the JVM heap memory since the Spark executor only handles data processing that fits the given time window. It also maintains Spark UMM regions as stable below the heap limitl see Figure 5b. In summary, it may be concluded that Spark backpressure is effective when handling non-intrusive memory applications, such as stateless.

### 5.2. Stateful-Based Application Performance Evaluation

This section presents two different assays. Section 5.2.1 shows the analysis of the Stateful SUMServer application without backpressure, and Section 5.2.1 demonstrates the analysis of the Stateful SUMServer application with backpressure. Finally, Section 5.2.3 presents, in detail, a GC comparison analysis.

#### 5.2.1. Non-Backpressure Assay

This section presents a native-based performance analysis of Spark for data-intensive processing pipelines without using a backpressure mechanism. This experiment aims to verify potential memory-based issues and their related performance impacts on the Stateful SUMServer application.

**Figure 6:**
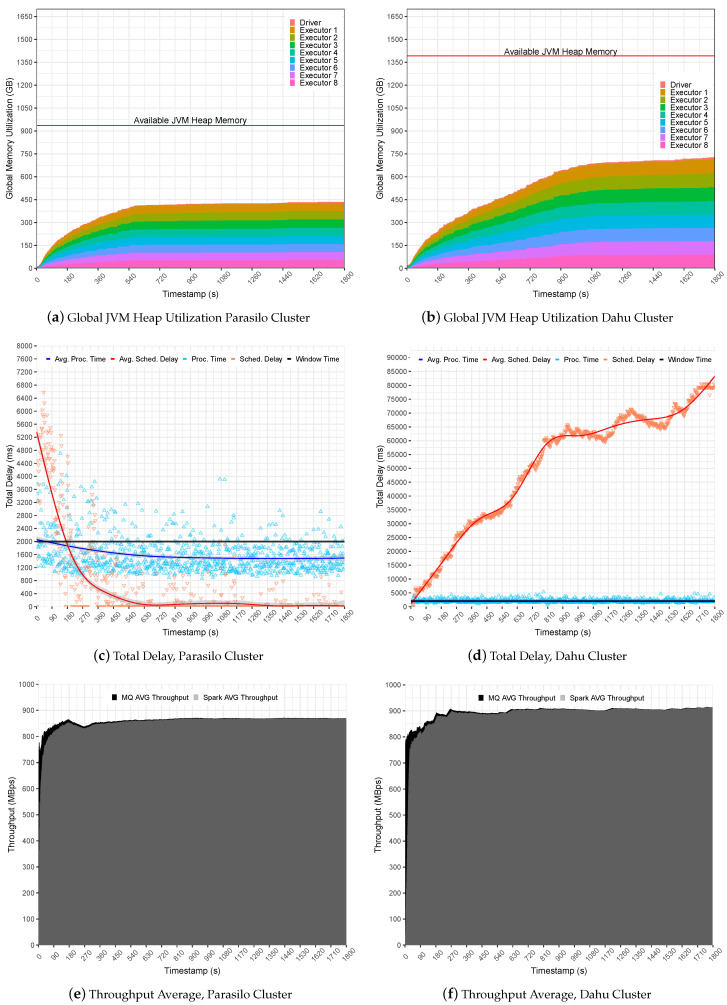
Stateful SumServer Application Without Backpressure—Pipeline 1.

Figure 6 summarizes the experiments for the SUMServer Stateful application over Pipeline 1. Figure 6a (Parasilo) and Figure 6b (Dahu) presents a global JVM heap memory utilization in GB. These figures are stacked and aim to present the total memory used per executor and driver components over time in seconds. Still, Figure 6c (Parasilo) and Figure 6d (Dahu) present the total delay related to PT, SD metrics, and their respective averages in ms over time in seconds. Finally, Figure 6e (Parasilo) and Figure 6f (Dahu) present the aggregated throughput average for data sent by the MQ and processed by Spark.

The experiments in Pipeline 1 demonstrate how simple changes in the application life cycle may affect the overall SP pipeline performance. Figure 6a presents a regular use of the JVM heap memory in the Parasilo cluster. It demonstrates that almost 50% of allocated heap memory is free to be used for Executors and Driver instances.

This occurs because Spark completely takes the incoming data, and there are no data being cached in heap memory, leading to a regular and stable use of the heap memory. The PT and SD reinforce this assumption since the metrics were intensive at the beginning due to high data ingestion but steady when data processing was faster than data sending to the MQs. In this case, the PT stabilized below the defined time window (1585 ms on average), and the SD did not present any intrusiveness (519 ms on average), indicating an under utilization of resources; see Figure 6c.

In the opposite direction, see Figure 6b for cluster Dahu. The JVM heap memory utilization grows over time, and the heap is not uniform, leading to a potentially unstable processing scenario. This happens because Spark Streaming queues data in memory for later processing, and this behavior is reflected in heap memory utilization.

In this case, data ingestion is slightly faster than the processing capacity for a single time window. The PT, for instance, reached up to 2094 ms on average; see Figure 6d (Grenoble). Unfortunately, case Spark cannot keep PT alongside the window time. The SD will keep growing until the JVM heap memory gets full, overloading Spark UMM execution, storage, and user memory regions in the JVM. It is important to mention unpredicted data ingestion, as presented in the Dahu cluster, which reached an SD of 50,679 ms on average, which may lead the application to an unstable processing scenario. As a consequence, the system falls behind due to OOM at JVM heap memory.

Finally, it is possible to observe that Figure 6e,f presents a similar average throughput, slightly better in the Dahu cluster. Although the Dahu cluster allowed for a promising throughput gain for long-term applications, the average PT obtained overpasses the window time, demonstrating that the system starts to be under pressure. Table 6 summarizes the obtained performance indicators for the evaluated scenario.

**Figure 7:**
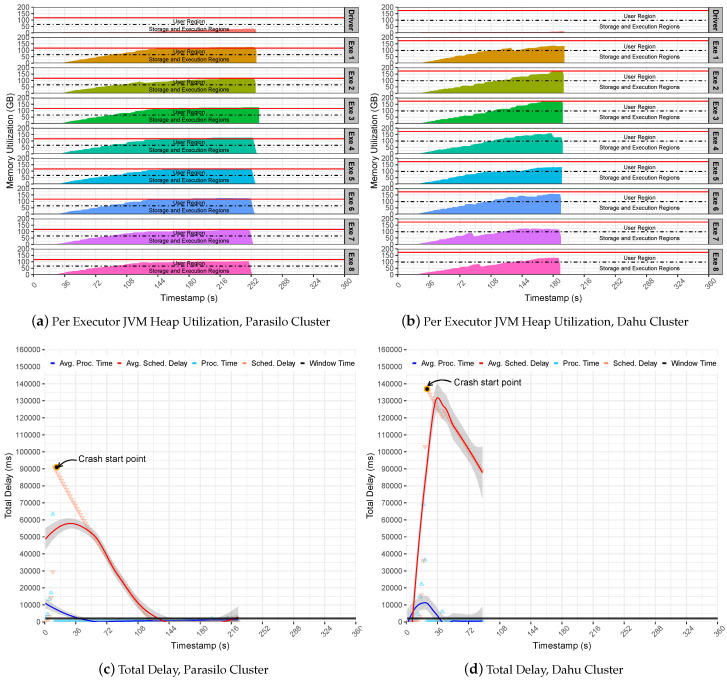
Stateful SumServer Application Without Backpressure—Pipeline 2.

The second set of experiments uses Pipeline 2 to evaluate how SS reacts to the data-intensive pipelines. Figure 7 summarizes the experiments for the SUMServer Stateful application over Pipeline 2. Figure 7a (Parasilo) and Figure 7b (Dahu) present the per node JVM heap utilization in Parasilo and Dahu clusters. The figures aim to present the total memory used per executor and driver components over time in seconds. Still, Figure 7c,d presents the total delay related to PT, SD metrics, and their respective averages in ms over time in seconds.

Figure 7 revealed limitations related to the internals of SS and its incapacity to support data management and memory coordination for a sudden surge of data. As we can see in Figure 7c (Parasilo) and Figure 7d (Dahu), the PT is at least five times greater than the time window. Although this behavior helps to increase the data throughput, the Spark UMM execution and storage memory will be quickly overwhelmed with data. Thus, as UMM has priority during processing tasks, it can disturb memory-boring operations at the UMM level and raise data block exceptions. This means that data will be lost and performance issues will be imminent. In fact, Table 7 presents exactly this behavior. In such a case, after data ingestion peaks, several exceptions happened, and the loss of data was noticed.

Consequently, the SD increases quickly since the Spark cluster cannot handle the incoming data properly. This may lead to a crash scenario since too much data are waiting to be processed: more than one minute in Parasilo and more than two minutes in the Dahu cluster.

It is possible to see in Figure 7a,b memory regions (execution and storage) from executors’ getting full of data and spilling data to user memory. In such a case, the executors may start to evict data blocks from memory by using the LRU algorithm to free some space for storing the new RDD blocks.

Furthermore, the sudden surges of data may congest Spark Block Manager at the driver’s daemon. This overwhelms the driver with metadata from RDD block, states, shuffle operations, data caching, and processing references. Consequently, the communication between the executors process will take longer, reproducing an invalid state of the system, and incurring application processing failures at any execution point. See the Start Crash Points in Figure 7c (Rennes) and Figure 7d (Grenoble).

These points represent an internal noise at the system level because receivers continuously receive incoming data and perform data processing tasks. In contrast, the PT decreases until 0, while SD follows the same direction. In summary, if the incoming data are not under control and PT is not steady, the eviction process becomes inevitable. In such a case, Spark cannot be considered stable anymore, losing data and collapsing completely due to the OOM issue at JVM heap memory.

Finally, the preliminary results indicated that Spark could not handle a sudden data surge. Still, the need for a system or feature to help in controlling data processing for SP applications is noticeable. This is the point where Spark backpressure comes in. It was designed to handle the sudden surge of data and to keep processing stable. In such a case, the following section evaluates how Spark handles data-intensive pipelines by applying the backpressure mechanism.

#### 5.2.2. Backpressure-Based Assay

This section presents a backpressure-based performance analysis. This evaluation investigates how Spark reacts to data-intensive processing pipelines using the backpressure mechanism and related components. The first step of this analysis evaluates the use of the *spark.streaming.backpressure.initialRate* property and its related impact on the application’s performance.

The *spark.streaming.backpressure.initialRate* property helps Spark backpressure to define the initial maximum receiving rate of the executors. The experiments were conducted using the Stateful SUMServer application on Dahu and Parasilo clusters over Pipeline 2; observe the pipeline configuration in Table 5 and the Spark configuration in Table 4.

**Figure 8:**
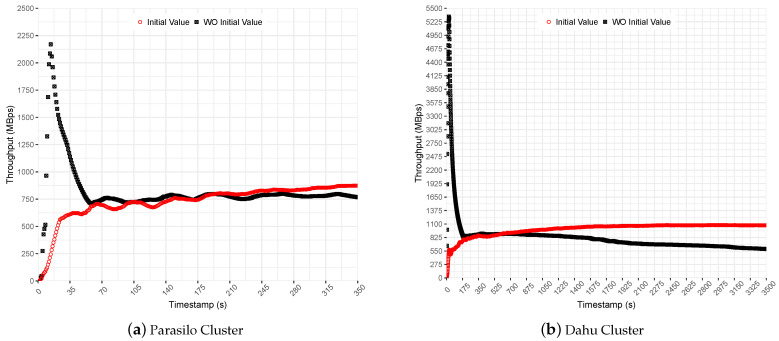
Backpressure Initial Rate Feature Comparison for Stateful SUMServer Application—Pipeline 2.

Figure 8 shows Spark’s throughput for Stateful SUMServer application in MBps for Dahu and Parasilo clusters. Each figure presents the application performance with and without the initial rate property configured. The initial rate value was set to allow up to 2000 records per executor. It represents a small value that introduces a slow start to help the PID algorithm in the preliminary measurements.

In the experiments performed without an initial value set for the backpressure, it is possible to observe a sudden surge of data at the beginning of execution; see Figure 8b. Although it presented a high throughput for a short time, it ramped the SD up quickly. Thus, even backpressure keeps processing rates under control. Spark accumulated a huge amount of data in the executors’ cache, requiring a considerable effort from PID backpressure to stabilize the processing rates on the fly; see the obtained average throughput for this comparison in Table 8.

Then, backpressure identified that the processing rate was greater than the time window and suddenly decreased the number of events allowed to be processed per executor. This process is effective but time-consuming since the state remains in memory for a long time, degrading the performance over time.

In comparison, in the experiments performed with an initial value set for the backpressure, the initial rate slowed down the application’s processing at the beginning of execution. Although processing slows at the beginning, it helped the PID controller to adjust to steady processing rates; see the obtained throughput average (initial values) in Figure 8a,b.

Finally, the results indicate that initial values improved the performance to 14% in the Parasilo cluster and 82% in the Dahu cluster. The following section presents the backpressure evaluation for data-intensive SP pipelines. The following experiments used the Stateful SUMServer application with the backpressure *spark.streaming.backpressure.initialRate* feature enabled and set to 2000 records per executor.

**Figure 9:**
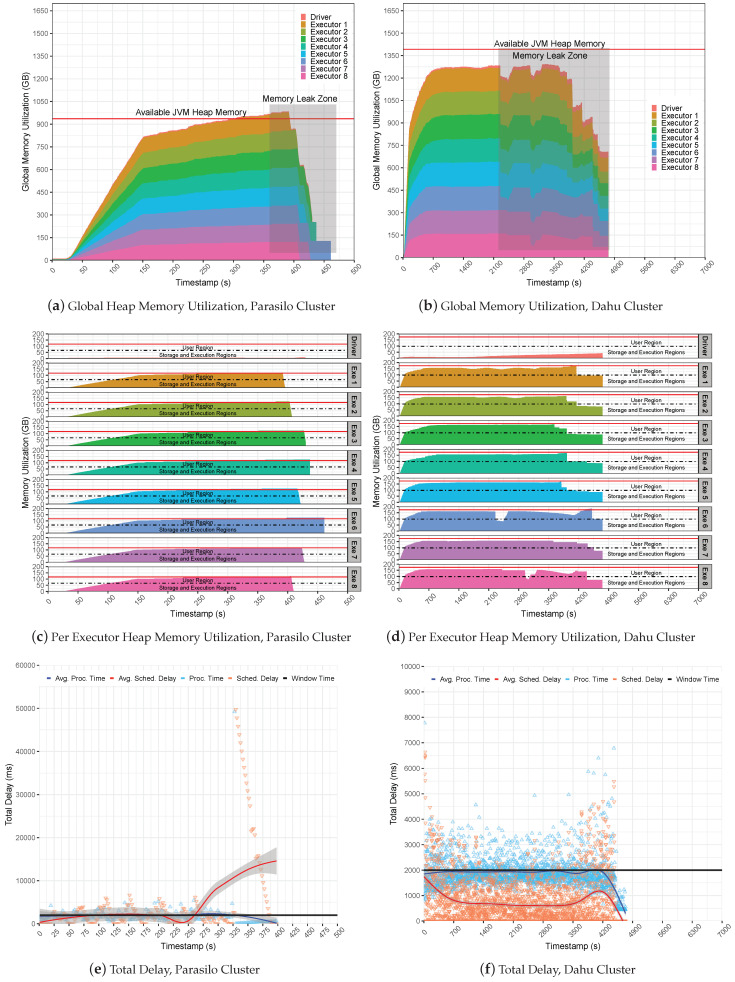
Stateful SumServer Application With Backpressure—Pipeline 2.

Figure 9 presents the global JVM heap memory utilization in GB for the Stateful SUMServer application in Pipeline 2 in Figure 9a (Parasilo) and Figure 9b (Dahu). The figures are stacked and aim to present the total memory used per executor and driver components over time in seconds. Figure 9c,d shows per-executor JVM heap utilization. Still, Figure 9e (Parasilo) and Figure 9f (Dahu) present the total delay, which shows the PT, SD metrics, and their respective averages over time in seconds.

It is possible to observe that backpressure failed to keep processing under control, leading to a crash issue in both clusters. This occurred due to a lack of management of incoming data at the Spark level, meaning that the MQ kept sending data to Spark receivers without any control. Internally, the backpressure took care of SD and PT metrics by managing executor processing rates to fit with the window time as presented in Figure 9e,f.

However, backpressure could not keep UMM healthy since Spark UMM execution has priority over the storage region, leading to a memory starvation condition. In this case, Spark keeps filling the storage region that tries to borrow space from the execution region; the user region will be used as a “backup” to avoid OOM issues since Spark was configured to use the on-heap data persistence. Thus, in the case that JVM gets full of data, a new stream of data and a set of old RDD blocks will be evicted from memory to allow for free space for new computation and storage needs. Thus, as the SUMServer can not compute their jobs due to data loss, the application falls behind, raising an issue known as Block Not Found Exception.

The eviction process may be observed in Figure 9a,b; observe the Memory Leak zone regions in the Figures. Still, it is possible to observe in depth the JVM heap utilization per executor in Figure 9c,d. Figure 9a reveals the exact moment of the OOM issue; look at second 220 and analyze the memory utilization of the executors. Still, it is possible to see them reaching the JVM capacity side by several SD oscillations before a full outage, as demonstrated in Figure 9e. Similarly, the Dahu cluster presents the eviction process at the time slice 2200, 3000, 3800 (s), and others, as observed in Figure 9f.

Such a situation may occur because the storage region at the executors gets full of data, then borrows space from the execution and fills it, and finally overloads the user region. Thus, if a single executor starts to fail, it can lead to a system crash that will result in several GC operations or data eviction from memory by the LRU strategy from Spark. Finally, it is possible to observe that small-sized or non-intensive applications may use backpressure without any problem since those scenarios do not push Spark to the limit.

#### 5.2.3. Garbage Collection Comparison Analysis

This section aims to understand how GC operations imply the performance of data-intensive SP scenarios. It presents a performance comparison of the stateful SUMServer application performed under stress conditions with and without Spark backpressure over Pipeline 2. The GC metrics were collected for both Spark driver and executor instances by setting the *spark.driver.extraJavaOptions* configuration parameter and the respective value *-XX:+PrintGCDetails -XX:+PrintGCDateStamps -Xloggc:file.log*. The obtained data were parsed through the *Universal GC Log Analyzer tool Gceasy* (GC Analyser tool: https://gceasy.io/). The Gceasy application provides the following GC processes metrics from the logs:

- Ergonomic: this is a JVM and garbage auto-tuning process that dynamically tunes the JVM heap size to meet the application needs with minimum pauses;
- Allocation Failure: this happens when there is not enough free space to create new objects;
- GCLocker Initiated GC: this process prevents GC operations when the JNI code is in a critical region. Thus, if GC is needed while a thread is in a critical region, then it will allow them to complete, i.e., call the corresponding release function;
- Metadata GC Threshold: this process happens when a configured meta-space size is smaller than the current system requirements;

These processes may lead the JVM to perform the following GC operations:

- Minor GC stats: this collects garbage from JVM spaces. Minor GC is always triggered when JVM cannot allocate space for new objects, e.g., one region getting full. Thus, the higher the allocation rate, the more frequently that Minor GC will be executed.
- Full acGC stats: this cleans the entire heap—all memory spaces.

Furthermore, we can also observe some performance indicators, such as:

- Throughput (%): the percentage of time spent processing real transactions vs. time spent in GC activity. A higher percentage indicates that GC overhead is low;
- Avg Pause GC Time (ms): this is the average amount of time taken by one Stop-the-World GC pause;
- Max Pause GC Time (ms): this is the maximum amount of time taken by one Stop-the-World GC to run;

Table 9 summarizes the sources of GC operations, the GC operations, and some performance indicators for the scenario Stateful SUMServer application without backpressure. Initially, it is possible to observe that the driver instance achieves almost 100% of Throughput in both environments. It demonstrates that JVM heap memory at the driver node is healthy and does not represent a bottleneck. This occurs because JVM heap memory utilization at the driver is under control, resulting in a lower number of GC operations and maintaining a low latency when operations are required. Although the driver is healthy, the executors will keep processing and storing data at a high level in memory, overloading the JVM heap.

Thus, the pressure on the JVM introduced some performance degradation. For instance, the throughput metric for the executor degraded by at least 55% for both environments compared to the scenario with (Table 10) and without (Table 9) backpressure enabled. It is possible to observe that the performance degradation is mainly related to the successive GC operations made in both scenarios. In addition, they mainly came from allocation failures that happen when there is not enough free memory space to create new objects in memory. Thus, if the system is not under control, minor GC events will continuously try to allocate space for new objects to keep processing stable. However, if the Eden region is getting full, a major GC event may occur, clearing the entire JVM heap.

Table 10 summarizes the sources of GC operations, the GC operations, and some performance indicators for the scenario running the Stateful SUMServer application with backpressure. Initially, it is possible to see the driver achieving 100% of *Throughput* in both environments, like presented before. The GC operations at the driver side increased since it must be more active than a scenario where Spark suddenly crashes.

Finally, the obtained results at the executors were slightly better than a non-backpressure scenario. Both applications were kept alive and stable for a longer time—more than 300 in Parasilo and more than 2000 in the Dahu cluster—and maintained a satisfactory throughput level near 100%. Furthermore, backpressure was quite suitable since Dahu decreased the number of GC operations by more than 30%.

At the same time, Parasilo maintained a similar number of operations but for a longer execution time.The use of backpressure decreased memory utilization since it controls the speed at which data have been unrolling in memory. In addition, GC spends less time swapping objects in memory, avoiding minor or full operations at heap memory and maximizing the applications’ performance.

## 6. Conclusions

It is well known that the available memory of computing systems is constantly increasing, allowing for in-memory data processing at a high scale. Moreover, in-memory data-intensive frameworks have been widely used to handle challenging problems in various domains, such as machine learning, graph computing, and SP.

The use of backpressure seems to guarantee a kind of application’s stability for non-intensive scenarios, i.e., receiving data as fast as Spark can process in a single time window, or for scenarios without processing with states. Thus, the application could achieve stable processing at a high throughput in controlled conditions. However, it is noticeable that backpressure shifts the task of buffering incoming data to the senders until the stream application processes them. This approach can fail in high-intensive scenarios, as demonstrated in this work. This happens due to a lack of management at the UMM controlled by the PID algorithm, which only handles the execution region during processing, leading to memory faults such as OOM issues due to high utilization from the storage region.

Furthermore, UMM storage and execution shares the same memory regions. Then, while backpressure is performing rate adjustments, the receivers will keep pushing data and filling memory up to the JVM heap memory limit, reducing the space for execution due to high storage needs. In such a context, a surge of data may generate high SD peaks that lead to a crash issue and unhealthy conditions for processing since the system is not stable. Still, this means that the processing could not be made in a single time window because plenty of data are waiting in the executor cache to be later processed, letting the JVM heap memory on pressure at the executor side. In addition, when the Spark needs to evict data from memory, the execution has priority over the storage region, incurring data loss in intensive processing scenarios.

The needed time to evict data blocks is relative and really depends on the cluster capacity and processing performance. Still, several borrowing operations at the UMM level may lead to multiple entire GC operations to clean up RDD objects from JVM heap memory. Still, the cleaning operations are critical for processing because some old states may be required in the current processing steps. Thus, Spark will fail if a block is already evicted from the executors’ cache.

Finally, as far as we can see, the state of the art does not present a solution for this problem. The solutions relied upon small and medium-sized changes in the core of SP frameworks, such as a new memory manager, a new eviction policy, or improvements based on batching or GC counters. However, this study unveils current memory management issues, specifically the data caching operation at the JVM heap. This issue affects not only SS but all other SP systems that rely on JVM for processing. In addition, it is possible to look forward to future work, such as: providing a global coordination mechanism at upstream components to balance data forwarding based on JVM cache utilization policies; modifying the PID solution to manage data caching alongside an execution strategy; providing new data eviction models that track not only recent data block utilization but also data dependency.

## Figures and Tables

**Table 1:** Related Work: detailed overview.

**Ref.**	**Problem**	**Related Issues** **and Concerns**	**Processing** **Style**	**Agnostic** **Solution**	**Memory** **Coordination**	**Data** **Persistence**	**Solution**	**Intrusiveness**	**Upstream** **Management**
[22]	End-to-end-latency	Low throughput Data loss	Not specified	No	UMM	Not specified	Adaptive batch sizing solution with queue batching controller	Spark-core	No
[23]	Memory shortage	High scheduling delay Insufficient in-memory Spark’s low responsiveness	Stateful	No	UMM	Memory only	Data-driven latency controller with data shedding strategy	Spark-core	No
[24]	State checkpointing Latency	Low throughput Checkpoint latency Data loss Resource exhaustion	Stateful	No	UMM	Memory and Disk	Backpressure from Spark with Feedback Controller	Spark-core	No
[15]	Network latency	Load imbalance Resource exhaustion	Stateful	No	Flink over JVM	Memory and Disk	Adaptive batching with memory-based data scheduling	Flink-core	Yes
[21]	Memory shortage	Low throughput Checkpoint latency Data loss Resource exhaustion	Stateful	No	Flink over JVM	Memory and Disk	Backpressure mechanism with adaptive parallelism	Flink-core	No
[20]	Inefficient memory management	OOM crashes Data loss Resource exhaustion	Stateless	No	UMM	Memory and Disk	Application semantics GC-based memory manager and orchestrator for big data applications	Spark-core	No

**Table 2:** Spark Performance Counters.

Performance Counters	Definition
Time	The timestamp of the current batch interval that just finished;
Events	The number of records that were processed in the current batch;
Processing Time (PT)	Time in ms it took for the job to complete;
Scheduling Delay (SD)	The time in ms that the job spent in the scheduling queue;

**Table 3:** Software Stack.

Operating System Debian 9, Kernel 4.9.0-11 amd64
Hadoop 3.1.2
Spark Streaming 2.4.3
Java 1.8.081
Scala 2.13
OpenMpi 4.0.1
ZeroMQ 3.1.1

**Table 4:** Spark Configuration.

Parameters	Rennes Parasilo Cluster	Grenoble Dahu Cluster
Window (Batch Interval)	2000 ms	2000 ms
Block Interval	400 ms	400 ms
Concurrent DAGs	1	1
Spark Parallelism	default	default
#Driver Instances	1	1
#Executors instances	8	8
#Receivers per Executor	1	1
Main Memory per Node	128 GB	192 GB
Driver JVM Heap Memory	117 GB	174 GB
Executor JVM Heap Memory	117 GB	174 GB
Executor UMM Storage Region	33 GB	49 GB
Executors Global Storage Region Memory	264 GB	392 GB
Executors Global JVM Heap Memory	936 GB	1392 GB
Cores per Executors (HT)	32	64
#Total Cores in the Spark Cluster	256	512
JVM Memory Schema	On-heap	On-heap
GC Type	G1GC	G1GC

**Table 5:** Pipeline Configurations.

Cod.	Size	Data Sources	MQs Nodes	Driver Nodes	Executors (One per Worker Node)
Pipeline 1	Soft	8	1	1	8
Pipeline 2	High	8	8	1	8

**Table 6:** Performance Indicators of Stateful SUMServer Application—Pipeline 1 without Backpressure.

Metrics	Parasilo	Dahu
AVG Th (MBps)	870	918
AVG PT (ms)	1585	2094
AVG SD (ms)	519	50,679
AVG Proc. Events	95,051	100,279

**Table 7:** Performance Crashing Indicators of Stateful SUMServer Application—Pipeline 2 without Backpressure.

Metrics	Parasilo	Dahu
MAX PT (ms)	63,371	69,044
MAX SD (ms)	90,792	137,480
Crashing Start Time (s)	14	26

**Table 8:** Backpressure Initial Value Comparison.

	Initial Value Set Average Throughput (MBps)	Initial Value Not Set Average Throughput (MBps)
Parasilo	874	764
Dahu	1076	590

**Table 9:** GC Statistics for Stateful SUMServer Application Without Backpressure—Pipeline 2.

	Dahu Cluster		Parasilo Cluster	
	**GC Events** **Driver**	**GC Events** **Executors**	**GC Events** **Driver**	**GC Events** **Executors**
Ergonomics	0	133	0	19
Allocation Failure	13	1392	36	184
GCLocker Initiated GC	6	25	0	9
Metadata GC Threshold	0	22	3	2
Total	19	1572	39	214
	GC Operations			
Minor GC stats	16	1428	36	194
Full GC stats	3	144	3	20
Total	19	1572	39	214
	Performance Indicators			
Throughput %	99	45	99	45
Avg Pause GC Time (ms)	34	835	57	591
Max Pause GC Time (ms)	90	10,184	160	7920

**Table 10:** GC Statistics’ for Stateful SUMServer Application With Backpressure over Pipeline 2.

	Dahu Cluster		Parasilo Cluster	
	**GC Events** **Driver**	**GC Events** **Executors**	**GC Events** **Driver**	**GC Events** **Executors**
Ergonomics	1	125	0	38
Allocation Failure	725	1045	95	152
GCLocker Initiated GC	6	18	6	0
Metadata GC Threshold	1	32	0	30
Total	733	1220	101	220
	GC operations			
Minor GC stats	731	1079	98	167
Full GC stats	6	141	3	53
Total	737	1220	101	220
	Performance Indicators			
Throughput %	100	99	100	95
Avg Pause GC Time (ms)	26	338	21	238
Max Pause GC Time (ms)	300	1854	90	755

## Data Availability

Not applicable.

## Abbreviations

The following abbreviations are used in this manuscript:

API	Application Programming Interface
CPU	Central Processing Unit
DAG	Direct Acyclic Graph
HDFS	Hadoop Distributed File System
GC	Garbage Collection
JVM	Java Virtual Machine
JNI	Java Native Interface
LRU	Least Recently Used
MPI	Message Passing Interface
MQ	Message Queue
OOM	Out Of Memory
OS	Operating System
PID	Proportional-Integral-Derivative
PT	Processing Time
RAM	Random Access Memory
RAMFS	Random Access Memory File System
RDD	Resilient Distributed Datasets
RC	Rate Controller
RE	Rate Estimator
RL	Rate Limiter
SMM	Static Memory Manager
SD	Scheduling Delay
SP	Stream Processing
SS	Spark Streaming
UMM	Unified Memory Management
